# Global Transcriptomic Analyses Provide New Insight into the Molecular Mechanisms of Endocarp Formation and Development in Iron Walnut (*Juglans sigillata* Dode)

**DOI:** 10.3390/ijms24076543

**Published:** 2023-03-31

**Authors:** Anmin Yu, Hanyu Zou, Ping Li, Xiaowei Yao, Jiayu Guo, Rui Sun, Gaosheng Wang, Xueliang Xi, Aizhong Liu

**Affiliations:** 1 Key Laboratory for Forest Resources Conservation and Utilization in the Southwest Mountains of China, Ministry of Education, Southwest Forestry University, Kunming 650224, China; 2 Yunnan Academy of Forestry and Grassland, Kunming 650201, China

**Keywords:** endocarp, formation, lignification, iron walnut, RNA-seq

## Abstract

Iron walnut (*Juglans sigillata* Dode) is a native species in southwestern China that exhibits variation in both fruit morphology and shell thickness. However, the underlying molecular processes controlling hardened endocarp development in walnut has not yet been reported. Here, we generated transcriptional profiles of iron walnut endocarp at three developmental stages using “Dapao”, the most common commercial variety. Using pairwise comparisons between these three stages, a total of 8555 non-redundant differentially expressed genes (DEGs) were identified, and more than one-half of the total DEGs exhibited significant differential expression in stage I as compared with stage II or stage III, suggesting that the first stage may ultimately determine the final characteristics of the mature walnut shell. Furthermore, in the clustering analysis of the above DEGs, 3682, 2349, and 2388 genes exhibited the highest expression in stages I, II, and III, respectively. GO enrichment analysis demonstrated that the major transcriptional variation among the three developmental stages was caused by differences in cell growth, plant hormones, metabolic process, and phenylpropanoid metabolism. Namely, using the tissue-specific expression analysis and a gene co-expression network, we identified MADS-box transcription factor JsiFBP2 and bHLH transcription factor JsibHLH94 as candidate regulators of endocarp formation in the early stage, and JsiNAC56 and JsiMYB78 might play key roles in regulating the lignification process of endocarp in the late stage. This study provides useful information for further research to dissect the molecular mechanisms governing the shell formation and development of iron walnut.

## 1. Introduction

Walnuts are a commercial crop valued worldwide for their high nutritional value, including unsaturated fatty acids, proteins, and vitamins [1]. As a native species to southwestern China, the iron walnut (*Juglans sigillata* Dode) is widely cultivated in the Yunnan, Guizhou, and Sichuan provinces [2]. This species, however, is not considered to be the best quality walnut due to varietal complexity, variable nut size, and inconsistent shell thickness.

Shells are an essential component of the fruiting body anatomy of walnuts as they provide a protective layer around the kernel and protect the seed from environmental stress [3]. Lignin is the main chemical component of the walnut shell, contributing to its hardness, stiffness, and strength [4]. With hardened endocarps encased in fleshy mesocarps, walnuts, similar to many economically important crops such as almond, peach, hawthorn (*Crataegus* spp.), and pomegranate (*Punica granatum* L.), are drupes [5]. Lignified endocarp, which grows out of the inner layer of the ovary, forms the familiar hardened shell of walnuts [6]. Shell development starts with the formation of parenchymal cells, then secondary cell walls are deposited on the primary walls after the cessation of cell division and expansion, and finally, the parenchymal cells are differentiated into polylobate stone cells [3]. The molecular mechanisms driving stone cell formation and endocarp hardening have only received limited studies. Fundamental questions remain about the genes underlying the formation of hardened shells in walnuts and whether endocarp tissue differentiation is controlled in the same way as endocarp B layer (enb) development in Arabidopsis or xylem cell differentiation program in poplar [5,7]. Understanding the processes governing shell thickness has direct commercial relevance as it impacts the yield and quality of the kernel, as observed in oil palm (*Elaeis guineensis*) [8].

Recent progress in genome sequencing of Juglandaceae species such as *Juglans regia*, pecan (*Carya illinoinensis*), and Chinese hickory (*C. cathayensis*) offers exciting new opportunities for functional genomics research on related species such as walnuts [2,9,10]. While the structural development and chemical composition of walnut shells are well-studied, little is known about the molecular regulation of shell thickness [11]. Understanding the genetic regulation of shell thickness is of great value for the improvement of nut yield and quality. Several genes related to secondary wall deposition have been shown to play important roles during the formation of stone cells in pear (*Pyrus pyrifolia*), peach (*Prunus persica*), and Macadamia (*Macadamia integrifolia*) [12,13,14]. NAC and MYB transcription factor families (TFs) are known to regulate secondary cell wall formation in woody tissues. In pear fruits, a NAC gene, *PbrNSC*, was reported to be functionally equivalent to Arabidopsis *NST3* (*NAC SECONDARY WALL THICKENING PROMOTING FACTOR 3*), which could transcriptionally activate multiple target genes involved in secondary cell wall formation [15]. Moreover, PbrMYB169 and PbrmiR397 have been verified to be involved in stone cell formation via the regulation of lignification [16,17]. In addition, several genes related to endocarp determination have been identified in Macadamia, oil palm, peach, and Arabidopsis, including the MADS-box genes *SHP1* (*SHATTERPROOF 1*), *SHP2*, *STK* (*SEEDSTICK*), and the basic helix-loop-helix genes *ALCATRAZ* (*ALC*) and *INDEHISCENT* (*IND*) [13,14,18,19]. These genes promote endocarp differentiation, hence causing stone hardening in peach and dehiscence zone formation in Arabidopsis.

Using transcriptome sequencing of endocarp tissues collected at three key developmental stages in iron walnut, we aim to improve our understanding of the molecular underpinnings of shell formation and development. We identified the core genes related to these processes using analyses of tissue-specific expression and gene expression patterns during shell development. Finally, we validated eight of these core genes using qRT-PCR. Our analyses reveal genes and TFs related to endocarp cellular identity and lignification and provide novel insights into the regulatory mechanisms dictating the dynamic and coordinated cellular events for cell division, proliferation, and differentiation in fruit with a hardened endocarp.

## 2. Results

### 2.1. Morphological and Weight Changes during Iron Walnut Endocarp Development

**Figure 1:**
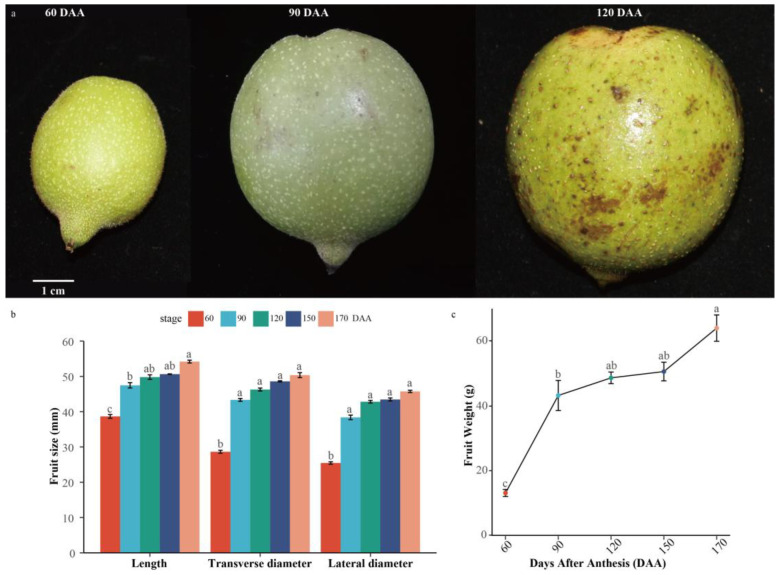
Fruit morphological characteristics during iron walnut development. (**a**) Morphology of iron walnut fruits at the rapid growth period (0–60 DAA), the endocarp hardening period (61–90 DAA), and the fruit maturation period (91–170 DAA). (**b**) The length, transverse diameter, and lateral diameter of fresh iron walnut fruit from 60 to 170 days after anthesis (DAA). (**c**) The weight of fresh iron walnut fruit from 60 to 170 DAA. Different letters indicate significant differences.

We investigated the morphological changes of the “Dapao” variety of iron walnut by comparing the fresh weight, length, and diameter of fruits from 60 to 170 days after anthesis (DAA) using five-time points (60, 90, 120, 150, and 170 DAA) for sampling. The complete walnut fruit developmental process can be generally divided into three different stages: the rapid growth period (stage I, 0–60 DAA), the endocarp hardening period (stage II, 61–90 DAA), and the fruit maturation period (stage III, 91–170 DAA) (Figure 1a). From 60 to 170 DAA, the fresh walnut fruit weight increased 4.78-fold (from 13.10 ± 0.62 g to 62.69 ± 2.03 g) (Figure 1b). During the whole fruit developmental process, the fruit length, transverse diameter, and lateral diameter increased 1.40-fold (from 38.67 ± 0.52 mm to 54.19 ± 0.39 mm), 1.75-fold (from 28.64 ± 0.41 mm to 50.00 ± 0.87 mm), and 1.73-fold (from 25.45 ± 0.34 mm to 44.02 ± 0.67 mm), respectively (Figure 1a). The fresh fruit weight at 90 DAA was 62.41% of the fully mature fruit (Figure 1b). At the same time, the fruit length, transverse diameter, and lateral diameter at 90 DAA reached about 86.05–93.67% of the final fruit size (Figure 1a). Thus, 90 DAA (stage II) should be considered the key developmental stage of walnut.

### 2.2. Comparative Transcriptomic Analysis of Developing Endocarp in J. sigillate

**Figure 2:**
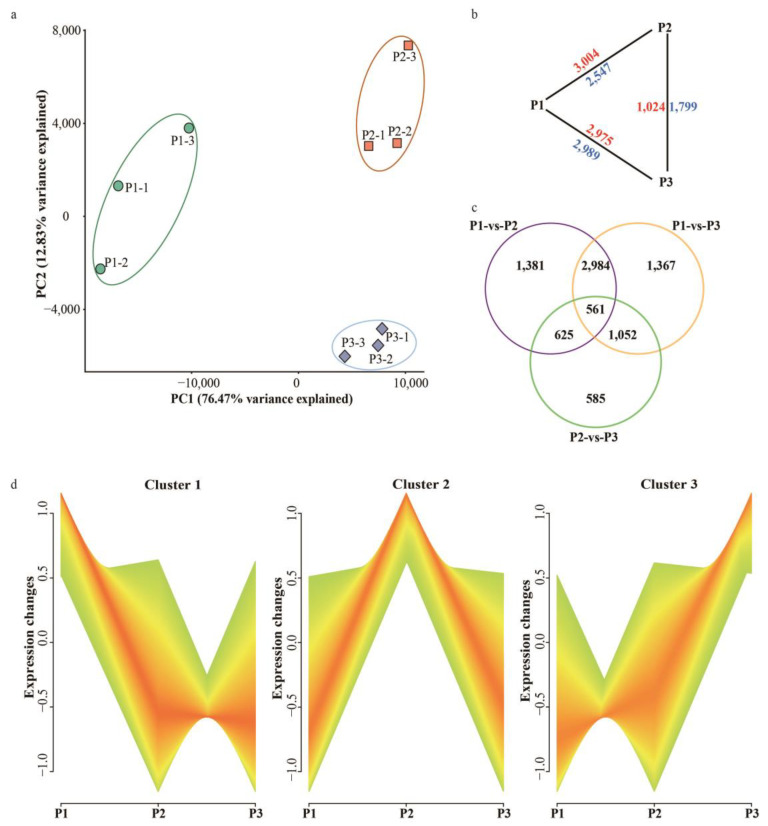
Principal component analysis (PCA) and hierarchical clustering dendrogram of the nine RNA sequencing libraries obtained from tissues collected at three development stages of the “Dapao” variety of iron walnut with three biological replicates for each. (**a**) Principal components one and two (PC1 and PC2) could explain 76.47% and 12.83% of the variance in the transcriptome samples. (**b**) The number of up- and down-regulated genes between the three different developmental stages of walnut. (**c**) Venn diagrams of the shared and unique genes among three compared pairs (P1 vs. P2, P1 vs. P3, P2 vs. P3). (**d**) Cluster dendrogram of the DEGs among three different developmental stages. P1: the endocarp of “Dapao” walnut at 60 DAA, P2: the endocarp of “Dapao” walnut at 90 DAA, P3: the endocarp of “Dapao” walnut at 120 DAA.

We used transcriptomic sequencing to examine the genetic mechanisms of endocarp development, and shell formation in “Dapao” walnuts using tissues collected 60, 90, and 120 DAA with three replicates of each from different fruits. These nine libraries were sequenced to an average of 7.37 Gb raw bases for each sample and with ~92.12% of the clean reads uniquely mapping to the *J. sigillate* genome (Table S1). Clean sequencing data can be found in the NCBI SRA database (accession number: PRJNA917462). Principal component analysis (PCA) of all nine samples was conducted, and two principal components, PC1 and PC2, could explain 76.47% and 12.83% of the overall variance, respectively (Figure 2a). For each developmental stage, the three replicates were spaced closely together, suggesting high consistency in the RNA-seq libraries (Figure 2b).

In total, we identified 8555 non-redundant DEGs using pairwise comparisons between the endocarp development stages (Tables S2–S4). Compared with stage I, a total of 2547 and 2989 DEGs in stages II and III, respectively, were significantly up-regulated, and 3004 and 2975 DEGs were significantly down-regulated (Figure 2c). Compared with stage II, 1799 and 1024 DEGs were up- and down-regulated, respectively, in stage III. Additionally, only 561 DEGs were detected across all comparisons (Figure 2c). These observations indicate that the first stage of walnut shell development is significantly different than the intermediate and last stages, and this may ultimately determine the final, realized characteristics of the mature walnut shell.

### 2.3. Clustered Analysis of Gene Expression during Shell Development

**Figure 3:**
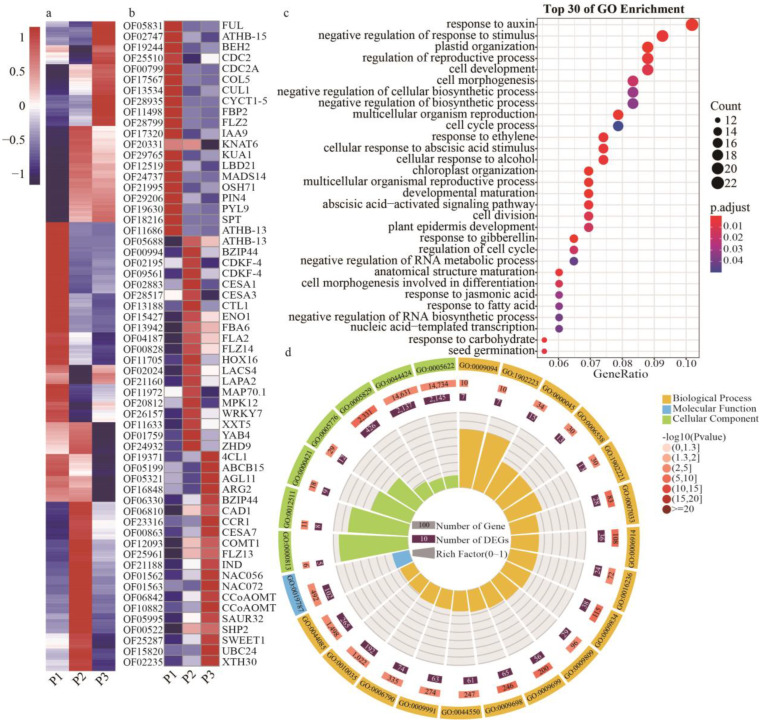
Analyses of gene expression patterns in developing shells of iron walnut “Dapao.” (**a**) The gene expression trends at three iron walnut shell developmental stages. Gene expression changes are expressed as log2-fold change (*y*-axis), and developmental stages are identified on the *x*-axis. (**b**) The gene expression trends of essential DEGs during three walnut shell developmental stages. (**c**) Enriched GO annotation of DEGs involved in the early developmental stage of walnut shells. (**d**) Enriched GO annotation of DEGs involved in the late developmental stage of walnut shells. P1: the endocarp of “Dapao” walnut at 60 DAA, P2: the endocarp of “Dapao” walnut at 90 DAA, P3: the endocarp of “Dapao” walnut at 120 DAA.

By clustering gene expression trends above DEGs, we identified gene sets showing significant differential expression during “Dapao” iron walnut shell development from stage I to stage III. At stages I, II, and III, 3682, 2349, and 2388 genes, respectively, exhibited the highest expression (Figure 2d, Table S5). GO enrichment analysis showed that genes involved in the cell cycle (GO:0007049), cell growth (GO:0016049), and cell division (GO:0051301) were significantly enriched with higher expression at stage I. These included *JsiCDC2 (cell division control protein 2 homolog*, *OF25510)*, *JsiCUL1 (Cullin-1*, *OF13534)*, and *JsiCYCT1-5 (Cyclin-T1-5, OF28935)* (Figure 3a,c). On the other hand, the GO terms of response to auxin (GO:0009733), abscisic acid−activated signaling pathway (GO:0009738), and response to jasmonic acid (GO:0009753) were enriched, including the genes *JsiIAA9 (OF17320)*, *JsiPYL9 (OF19630)*, and *JsiBEH2 (BES1/BZR1 homolog protein 2*, *OF19244)* (Figure 3b,c). In addition, several genes related to the regulation of the reproductive process (GO:2000241) and plant epidermis development (GO:0090558) were highly expressed in stage I, including *JsiSPT (Transcription factor SPATULA, OF18216)*, *JsiFUL (OF05831)*, *JsiATHB-15 (OF02747)*, *JsiOSH71 (OF21995)*, *JsiFBP2 (OF11498)*, and *JsiMADS14 (OF24737)* (Figure 3c). Furthermore, GO enrichment analysis of genes active in cluster II involved peptide metabolic process (GO:0006518), carbohydrate biosynthetic process (GO:0016051), methylation (GO:0032259), and protein folding (GO:0006457), including *JsiCTL1 (Chitinase-like protein 1*, *OF13188)*, *JsiCDKF-4 (Cyclin-dependent kinase F-4*, *OF09561)*, *JsiLACS4 (Long-chain acyl-CoA synthetase 4*, *OF02024)* (Figure S1). Additionally, these genes highly expressed in stage III were enriched with GO terms of ubiquitin-like protein transferase activity (GO:0019787), phenylpropanoid metabolic process (GO:0009698), and plant-type secondary cell wall biogenesis (GO:0009834) (Figure 3d), including *JsiSHP2 (OF00522)*, *JsiIND (OF21188)*, *JsiABCB15 (OF05199)*, *JsiNAC56 (OF01562)*, *JsiNAC072 (OF01563)*, *JsiCAD1 (OF06810)*, *JsiCOMT2 (OF20484)*, and *JsiUBC24 (OF15820)*. Together, these data demonstrated major transcriptional variations among the three developmental stages were caused by differences in cell growth, plant hormones, metabolic process, and phenylpropanoid metabolism.

### 2.4. Global Gene Expression Analyses Identify Endocarp-Specific Genes

**Figure 4:**
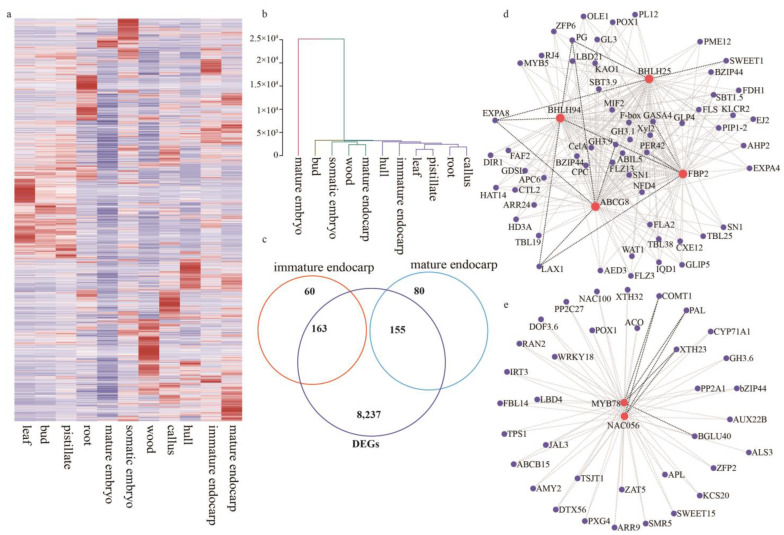
Identification of essential DEGs controlling walnut shell development. (**a**) Heatmap representation of transcription levels of tissue-specific genes across eleven different tissues (leaf, bud, pistillate, root, somatic embryo, mature embryo, wood, callus, hull, immature endocarp, and mature endocarp tissues). (**b**) Hierarchical clustering tree of all eleven tissues based on the gene transcript levels. (**c**) Venn diagrams of the shared and unique genes among immature endocarp, mature endocarp, and DEGs. (**d**) Predicted transcriptional regulatory network in the immature endocarp. The network is composed of essential TFs and their target genes. (**e**) Predicted transcriptional regulatory network in the mature endocarp. The central TFs related to immature and mature endocarp development are represented in red, and their target genes are represented in blue.

To further identify and characterize the endocarp tissue-specific genes, we investigated the comprehensive transcriptome dynamics of walnuts in eleven diverse tissues from different developmental stages encompassing the flower, fruit, and stem. Raw data were previously published [10] and downloaded from NCBI (SRS957431), then were mapped to the iron walnut reference genome, and the expression level of each transcript was calculated using fragments per kilobase of transcript length per million mapped reads (FPKM). Hierarchical clustering analyses of the above 11 tissues samples were conducted based on the gene expression level, and these tissues mainly clustered into four groups with the mature endocarp tissue clustering with wood, somatic embryo tissues, and the immature endocarp clustering with hull, leaf, pistillate, root, and callus (Figure 4a,b). These results indicate strikingly different gene expression patterns between mature and immature endocarp tissues. In addition, the mature embryo or bud did not cluster with any other tissues, suggesting their distinct transcriptional mechanisms. Furthermore, to identify candidate gene-specific expression in each tissue or stage, genes with expression values lower than 0.2 FPKM in one sample were removed, and a total of 15,100 genes were used for further examination using the SEGtool package. In total, 3040 genes were identified as tissue-specific expressed genes, including 223 and 235 genes in immature and mature endocarp, respectively (Figure 4c, Table S6). Then, we combined the endocarp-specific genes with the results of DEGs and identified 163 and 155 genes as specific to immature and mature endocarp, respectively (Figure S2). Among these DEGs, a total of 17 and 20 TFs were immature and mature endocarp tissue-specific, respectively (Figures S3 and S4). We used a co-expression analysis via GENIE3 to further characterize TFs associated with walnut shell formation and identity their potential target genes. At the immature stage, the co-expression network showed that *JsiFBP2 (FLORAL BINDING PROTEIN 2, OF11498)*, *JsibHLH25 (OF06682)*, *JsibHLH94 (OF18599)*, and *JsiABCG8 (OF26503)* shared the maximum number of nodes with target genes, including *JsiEXP8 (Expansin 8*, *OF27971)*, *JsiPG (Polygalacturonase*, *OF01947)*, *JsiLAX1 (LIKE Auxin 1*, *OF23724)*, and *JsiGH3.9 (Putative indole-3-acetic acid-amido synthetase*, *OF16813)* (Figure 4d, Table S7). This indicates they are essential factors in the formation of walnut shells. At the mature stage, *JsiMYB78 (OF02401)* and *JsiNAC56 (OF01562)* were co-expressed with the largest number of target genes, including *JsiXTH23 (Probable xyloglucan endotransglucosylase/hydrolase protein 23*, *OF15252)*, *JsiPAL (Phenylalanine ammonia-lyase*, *OF26774)*, *JsiBGLU40 (Beta-glucosidase 40*, *OF11621)*, *JsiCOMT1 (Caffeic acid 3-O-methyltransferase 1*, *OF12093)* (Figure 4e, Table S7). Furthermore, the cis-element analysis revealed that the *JsiEXP8 (OF27971)* and *JsiPG (OF01947)* promoters contained the CArG and E-box motifs, which are functional cis-element of FBP2 and BHLH proteins. In the promoter of *JsiXTH23 (OF15252)* and *JsiPAL (OF26774)*, we found the cis-element of NAC and MYB, secondary wall NAC binding element (SNBE), and AC elements (Figure S5). Hence, it is hypothesized that while the MADS-box genes *JsiFBP2* and *JsibHLH94* play important roles in controlling cell identity and development in the early endocarp formation stage, *JsiNAC56* and *JsiMYB78* control the thickness of the secondary cell wall during iron walnut endocarp development.

### 2.5. QRT-PCR Validation

**Figure 5:**
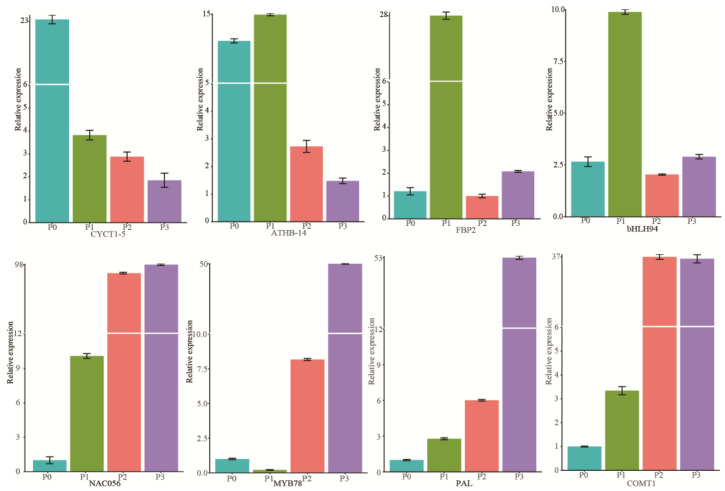
Validation of transcriptomic data using qRT-PCR. The *x*-axis represents the different stages of “Dapao” walnut shell development at 30, 60, 90, and 120 DAA. The *y*-axis represents the gene expression level, which is the 2^−∆∆ct^ value of qRT-PCR compared to the reference gene. Data are the means of three replicants. Error bars indicate SDs. P0: the endocarp of “Dapao” walnut at 30 DAA, P1: the endocarp of “Dapao” walnut at 60 DAA, P2: the endocarp of “Dapao” walnut at 90 DAA, P3: the endocarp of “Dapao” walnut at 120 DAA.

From the significant DEGs we identified, a total of eight genes were selected for further validation of the gene expression patterns in the endocarp of “Dapao” at four fruit development stages using qRT-PCR. The results indicated that the early-stage expression levels of four genes/TFs related to cell division or identity (*JsiCYCT1-5*, *JsiATHB-14*, *JsiFBP2*, and *JsibHLH94*) were higher than the middle or late development stages (Figure 5). Meanwhile, the expression levels of four TFs/genes involved in secondary cell wall formation, including *JsiNAC56*, *JsiMYB78*, *JsiPAL*, and *JsiCOMT1*, gradually increased through the developmental process. These results revealed that mainly genes involved with cell division and identity govern shell formation, and related genes showed higher expression levels in the early developmental stage. Then, cell differentiation and lignification began with lignin deposition in the endocarp, and the essential genes involved exhibited higher expression levels at the middle and late development stages. We observed that the relative gene expression levels between qRT-PCR and RNA-seq had a high correlation coefficient (Figure S6), indicating the transcriptome sequencing data was reliable.

## 3. Discussion

Previously, the underlying molecular processes controlling hardened endocarp development in drupes were unclear, despite their being vitally important commercial crops. Research on endocarp determination and differentiation in peaches revealed the process is similar to pod shatter or dehiscence in Arabidopsis [13]. It has also been shown that MADS-box transcription factors were required for floral organ identity, ovule identity, seed abscission, and endocarp lignification [20]. Floral development is regulated by the ABCDE model, and all of these genes, except A-class genes (*APETALA2*), belong to MADS-box TFs. In this model, B, C, and D class genes are essential for organ formation and development, and E class genes, such as *SEPALLATA* (*SEP*) and *JOINTLESS* (*J*), act as mediators of the higher-order protein complex [21]. The genes that control ovule identity were firstly isolated from *Petunia hybrida* and were named *FLORAL BINDING PROTEIN 7 (FBP7)* and *FBP11*, which encode MADS-box TFs [22]. In Arabidopsis, the MADS-box gene *SEEDSTICK* (*STK*, also known as *AGAMOUS-LIKE 11*) works as the ovule identity factor, which controls ovule identity, seed coat development, and lignified endocarp [23,24]. In rice, the orthologue of *FBP7*, *FBP11*, and *STK* is *OsMADS13*, which is a key regulator of ovule identity [25]. The function and expression of these MADS-box genes are conserved in higher plants. In oil palm, a homolog of *STK* was named as *Shell* gene, which is responsible for the shell-less phenotype [26]. Recently, in macadamia, MADS-box genes *MiTT16* and *MiSTK* have been identified as the major regulators during the formation of hard-shell in the macadamia fruit [14]. Here we found that the MADS-box genes *JsiFBP2 (OF11498)*, *JsiAP1C (OF11596)*, *JsiMADS14 (OF24737)*, and *JsiFUL (OF05831)* are highly expressed in the endocarp tissues of iron walnut, especially in the early stage (Figure 3b), which indicated their important roles in organ identity.

In addition, the bHLH-type proteins IND and SPT were found to mediate the proper pattern of lignin deposition during fruit dehiscence and seed dispersal in Arabidopsis, and there were controlled by the MADS-box proteins FUL and SHP [27]. In addition, IND was shown to interact with SPT proteins to regulate the formation of auxin minimum by controlling auxin transport [28]. The bHLH proteins usually interact with themselves or other proteins and act in homodimers and heterodimers. In *Chrysanthemum*, a bHLH protein CmHLB could promote lignin synthesis by forming a heterodimer with KNOX transcription factor CmKNAT7 [29]. Similarly, in upland cotton (*Gossypium hirsutum* L.), *GhbHLH18* bound to the promoter of *GhPER8* and activated the peroxidase-mediated lignin metabolism [30]. We found *JsiSPT (OF18216)* and *JsibHLH94 (OF18599)* were highly expressed in the first stage of endocarp development, but IND was highly expressed in the last stage (Figure 3b and Figure 4d). *SPT* is the first bHLH gene found to play an essential role in floral organogenesis, and previous research on *Solanum lycopersicum* found that the expression of *SPT* is restricted to the endocarp, and it is mainly expressed in the early stage of fruit development [31,32]. *IND* is considered a key regulator of unequal cell divisions in the fruit of Arabidopsis, and it is required for controlling the lignification of margin cells in Arabidopsis [33,34]. Our results suggest that *JsiSPT* and *JsiIND* mediate endocarp development and affect not only the first step of cell type identity and cell division but also cell differentiation in the iron walnut endocarp.

Considering our observations in both the transcriptome and morphological data, we confirmed that secondary cell wall biosynthesis and formation mainly occur in the middle and late stages. It has been previously shown that secondary cell wall formation is regulated through a multi-tier transcription network, including the first-layer master switches NAC TFs, second-layer master switches MYB TFs, some downstream regulators, and genes for lignin, cellulose and hemicellulose biosynthesis [35]. *NST1* (*NAC SECONDARY WALL THICKENING PROMOTING FACTOR1*) and *NST3*/*SND1* (*SECONDARY WALL ASSOCIATED NAC DOMAIN PROTEIN1*) were found to activate lignification of endocarp layers in peach and Arabidopsis [36]. In pumpkin (*Cucurbita pepo* L.), the *CpNST1* gene was responsible for the development of seed coat via the process of secondary cell wall formation of seed coat [37]. In this study, the expression patterns of *JsiNAC56 (OF01562)*, *JsiNAC072 (OF01563)*, *JsiMYB5 (OF16568)*, *JsiCAD1 (OF06810)*, and *JsiCOMT2 (OF20484)* were highest in the last stage of endocarp development (Figure 3b and Figure 4e). This suggests that the shell lignification and thickness formation of endocarps in drupes require the same pathway as that in wood, and hence this process is likely ancestral to angiosperm fruit development.

## 4. Materials and Methods

### 4.1. Plant Materials and Growth Conditions

We collected samples from the iron walnut cultivar “Dapao” in Guangming town, Dali Autonomous Prefecture, Yunnan Province, China. Fresh fruit samples were harvested at 30, 60, 90, 120, 150, and 170 DAA. Three replicates (three independent trees per replicate) were harvested at each time point, and at least five representative fruits were sampled from each tree. The fruits were washed with distilled water twice, and then the green husk was stripped cleanly. After separating the shell from the kernel, the samples were flash-frozen in liquid nitrogen and stored at −80 °C. The remaining samples were stored at 4 °C for walnut morphology data collection.

### 4.2. Transcriptome Sequencing and Differential Gene Expression Analyses

The shells of “Dapao” across three developmental stages were used for transcriptome sequence with three replicates from different fruit at each. Total RNA was extracted using an RNAprep pure Tissue Kit (Tiangen, Beijing, China). RNA quality and integrity were assessed using a NanoDrop 2000 spectrophotometer and an RNA 6000 Nano Kit for the Agilent 2100 Bioanalyzer (Agilent Technologies, Santa Clara, CA, USA). A total of nine libraries were sequenced on an Illumina Hiseq 2000 platform at the OE biotech company (Shanghai, China). The raw sequence data were filtered using the NGS QC Toolkit at default parameters to obtain high-quality reads. Then clean reads were mapped to the *J. sigillata* reference genome using HISAT2, and uniquely mapped reads were processed via StringTie to obtain the normal gene expression levels [38]. Correlation analysis, hierarchical clustering, and PCA were performed via the tidyverse package in R [39]. DESeq2 was used for differential expression analyses using the following conditions: differential expression fold |log 2 Fold Change| > 2 and significance *p*-value < 0.05 [40]. The gene expression patterns were generated by K-means methods using the Mfuzz package [41]. Heatmaps were created using the expression values with log2 (FPKM + 1) and Z-score normalization and visualized using the pheatmap package in R (https://cran.r-project.org/web/packages/pheatmap/index.html/ accessed on 10 August 2022).

### 4.3. Identification and Characterization of Tissue-Specific Genes

In order to identify important genes related to endocarp development, the RNA-seq data of 11 different walnut tissue samples were downloaded from the NCBI Sequence Read Archive (SRA) [10]. Clean reads were processed as described in Section 2.2. Then, the FPKM of each gene in all samples was calculated using Ballgown [42].

### 4.4. Gene Co-Expression Analysis and Cis-Element Predictions

Co-expression network analyses were performed through the R package GENIE3 based on the FPKM values of identified genes specifically expressed in immature and mature endocarp tissues [43]. Subsequently, the networks were visualized using Cytoscape (v.3.9.1) [44]. To further confirm the regulatory relationship between hub TFs and DEGs during walnut shell development, the cis-elements in the promoters of key genes were identified by the New PLACE (https://www.dna.affrc.go.jp/PLACE/?action=newplace accessed on 19 August 2022) [45]. All the key genes identified in this study were listed in Table S7, and genes can be found in the GenBank database through the accession numbers of *J. regia* and Arabidopsis

### 4.5. QRT-PCR

Walnut shell samples at 30, 60, 90, and 120 DAA obtained from fruits of “Dapao” were used for qRT-PCR analysis. Versus the samples used for RNA-seq, an additional fruit developmental time point (30 DAA) was added. The isolation of total RNA was performed using the RNAprep pure Tissue Kit (Tiangen, Beijing, China). To confirm the accuracy of transcriptomic sequencing, a total of eight DEGs were selected for qRT-PCR assays. *JsiRH17 (OF29618)* was chosen as the internal control using the NormFinder software [46]. Gene-specific primers were designed using Primer3 (version 0.4.0) and were presented in Table S8. Real-time PCRs were performed in a 96-well plate on a Bio-Rad CFX96 PCR system (Hercules, CA, USA). The real-time PCR analysis of each gene was performed using three biological and three technical replications, and relative gene expression levels were calculated using the 2^−ΔΔCt^ method [47].

## 5. Conclusions

This is the first study to identify essential genes involved in three stages of endocarp formation in iron walnut through RNA-seq analysis. We found 8555 DEGs with comparisons of expression between the three stages. In stages I, II, and III, respectively, we found higher expression of genes involved in cell development and plant hormone signal transduction pathway, biosynthesis of carbohydrates, and secondary cell wall. In addition, between these results combined with the tissue-specific analysis of eleven different walnut tissues, we identified 223 and 235 immature and mature endocarp-specific genes, respectively, and co-expression analysis revealed that *JsiFBP2* and *JsibHLH94* might be candidate regulators of endocarp formation, and *JsiNAC56* and *JsiMYB78* might play key roles in regulating the lignification process of the endocarp. This study revealed critical information about the molecular mechanisms of walnut endocarp development and identified worthwhile focal genetic sites for breeding high-quality walnut varieties. Given the mechanisms seem to be ancestral to angiosperms, this information should be generally useful for understanding the genetic underpinnings of all drupe fruit endocarp formation.

## Data Availability

Not applicable.

## Supplementary Materials

The following supporting information can be downloaded at: https://www.mdpi.com/article/10.3390/ijms24076543/s1.
