# Supplementary material for: Global Transcriptomic Analyses Provide New Insight into the Molecular Mechanisms of Endocarp Formation and Development in Iron Walnut (Juglans sigillata Dode)
Source: Int J Mol Sci. 2023 Mar 31;24(7):6543. doi: 10.3390/ijms24076543 (PMC10094949; doi:10.3390/ijms24076543)
Supplement: Supplementary file 1 [file ijms-24-06543-s001.zip › Figure S.pdf]

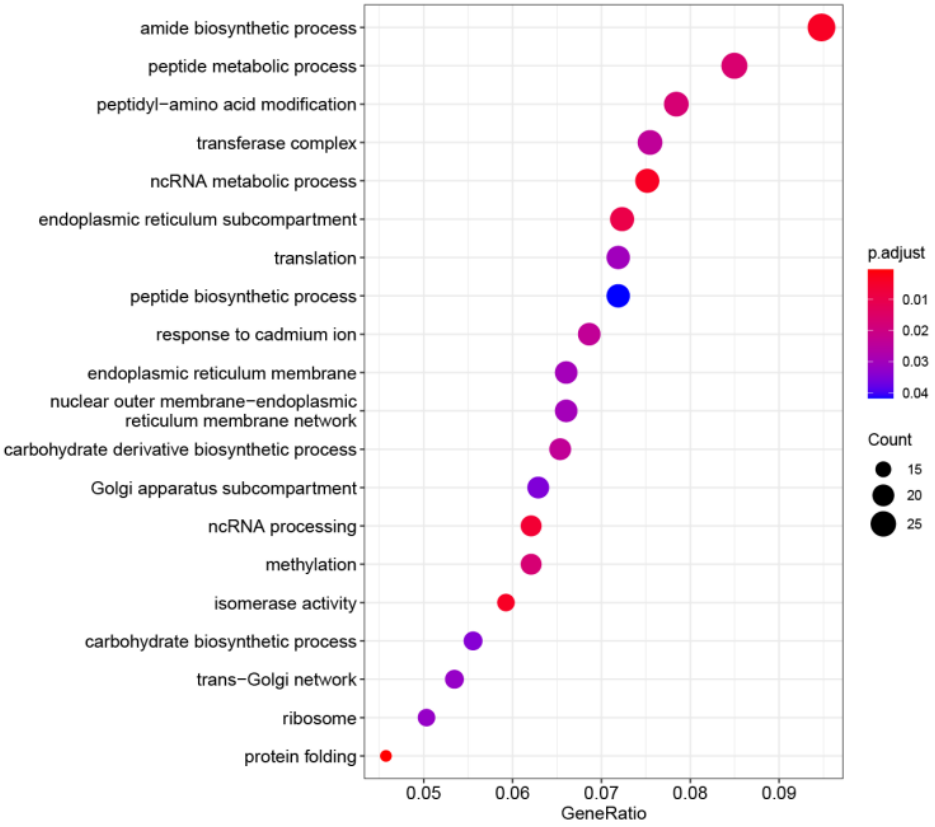

Figure S1 Enriched GO annotation of DEGs involved in the middle developmental stage of walnut shell.

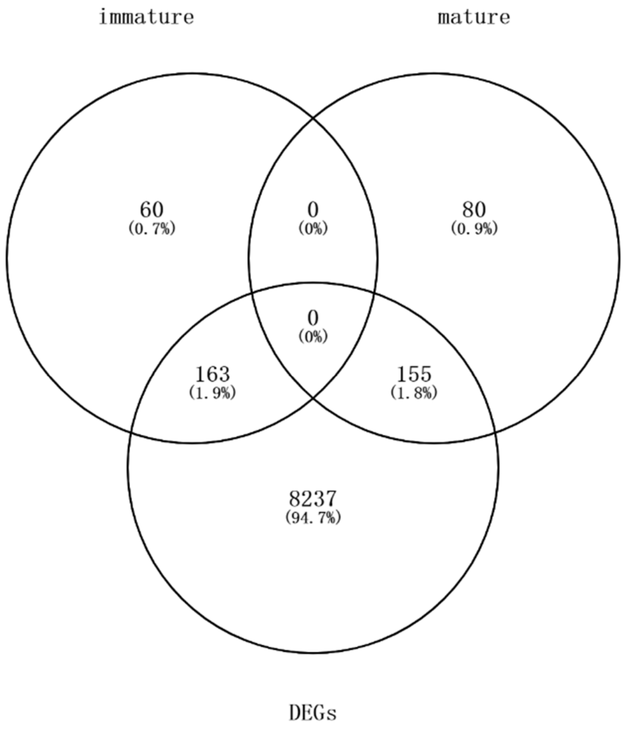

Figure S2 Identification of immature and mature endocarp specific DEGs.

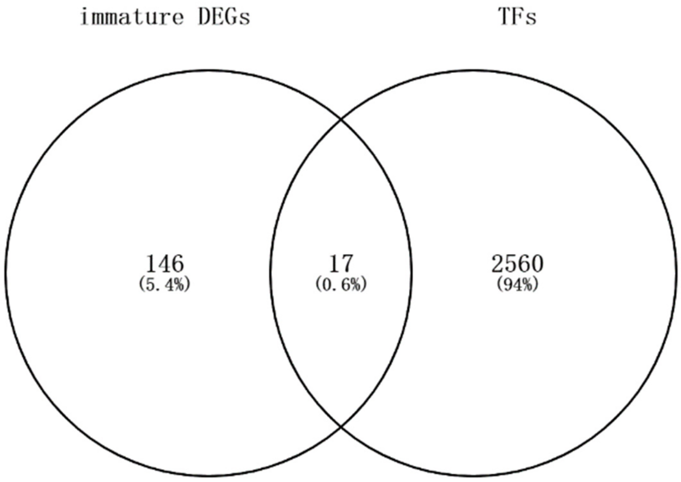

Figure S3 Identification of immature endocarp specific-TFs.

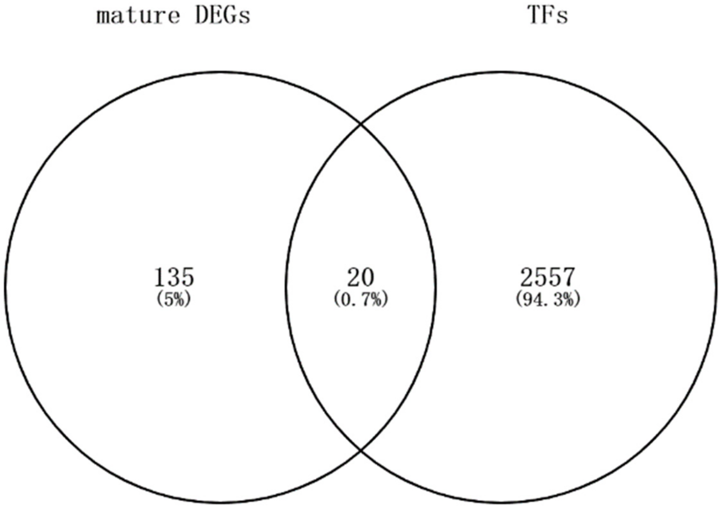

Figure S4 Identification of mature endocarp specific-TFs.

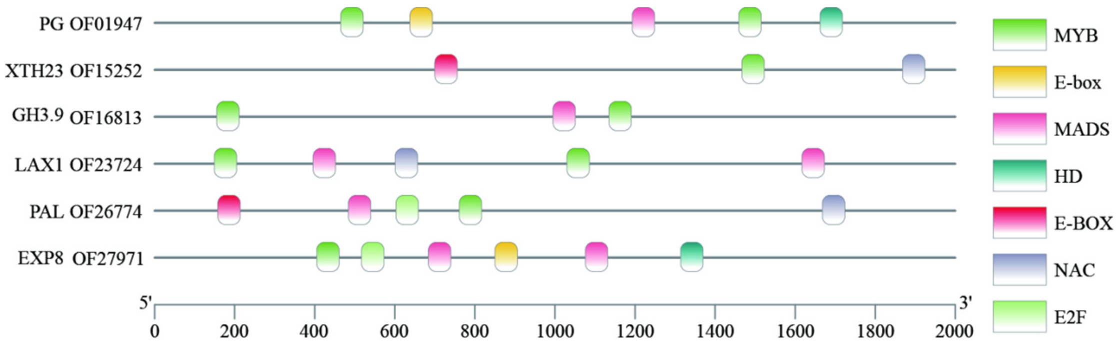

Figure S5 Cis-element analysis of essential genes associated with walnut shell

formation

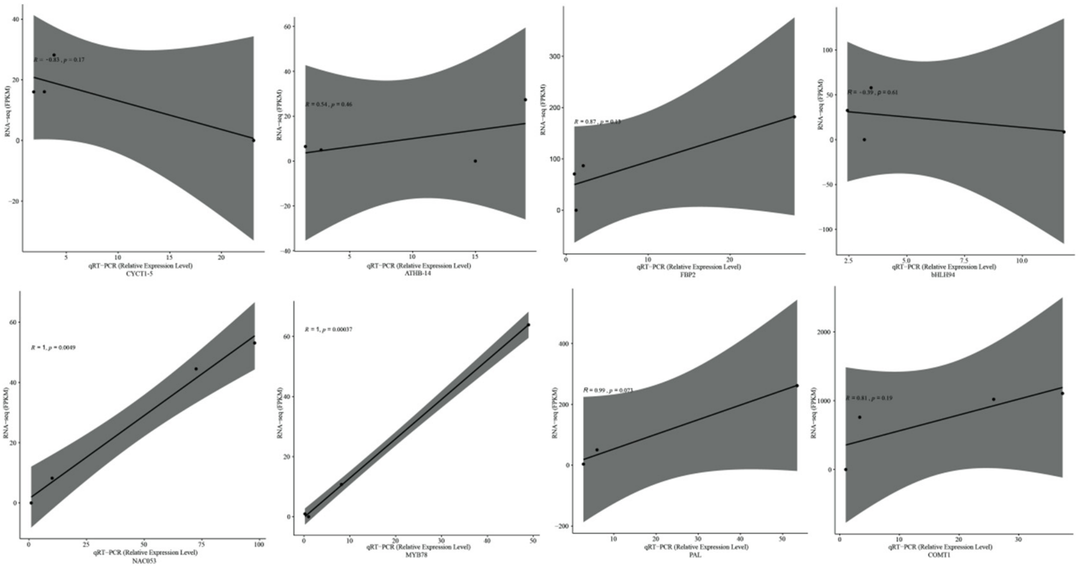

Figure S6 Correlation analysis for eight candidate genes between RNA-Seq and RT-qPCR.The X-axis represents the relative expression level of 8 candidate genes in RT-qPCR. The Y-axis shows the value of FPKM in RNA-Seq, which was used in calculated expression level.
